# Physiological Basis of Salt Stress Tolerance in a Landrace and a Commercial Variety of Sweet Pepper (*Capsicum annuum* L.)

**DOI:** 10.3390/plants9060795

**Published:** 2020-06-25

**Authors:** Pasquale Giorio, Valerio Cirillo, Martina Caramante, Marco Oliva, Gianpiero Guida, Accursio Venezia, Stefania Grillo, Albino Maggio, Rossella Albrizio

**Affiliations:** 1 National Research Council of Italy, Institute for Mediterranean Agricultural and Forestry Systems (CNR-ISAFOM), Ercolano, 80056 Naples, Italy; pasquale.giorio@cnr.it (P.G.); marcoliva@hotmail.it (M.O.); gprguida@gmail.com (G.G.); rossella.albrizio@cnr.it (R.A.); 2 Department of Agricultural Science, University of Napoli Federico II, Portici, 80055 Naples, Italy; almaggio@unina.it; 3 Council for Agricultural Research and Economics, Research Centre for Vegetable and Ornamental Crops (CREA-OF), Pontecagnano, 84098 Salerno, Italy; martina.caramante@gmail.com (M.C.); accursio.venezia@crea.gov.it (A.V.); 4 National Research Council of Italy, Institute of Biosciences and Bioresources (CNR-IBBR), Research Division Portici, 80055 Naples, Italy; grillo@unina.it

**Keywords:** chloride, sodium, photosynthesis, landraces, yield

## Abstract

Salt stress is one of the most impactful abiotic stresses that plants must cope with. Plants’ ability to tolerate salt stress relies on multiple mechanisms, which are associated with biomass and yield reductions. Sweet pepper is a salt-sensitive crop that in Mediterranean regions can be exposed to salt build-up in the root zone due to irrigation. Understanding the physiological mechanisms that plants activate to adapt to soil salinization is essential to develop breeding programs and agricultural practices that counteract this phenomenon and ultimately minimize yield reductions. With this aim, the physiological and productive performances of Quadrato D’Asti, a common commercial sweet pepper cultivar in Italy, and Cazzone Giallo, a landrace of the Campania region (Italy), were compared under different salt stress treatments. Quadrato D’Asti had higher tolerance to salt stress when compared to Cazzone Giallo in terms of yield, which was associated with higher leaf biomass vs. fruit ratio in the former. Ion accumulation and profiling between the two genoptypes revealed that Quadrato D’Asti was more efficient at excluding chloride from green tissues, allowing the maintenance of photosystem functionality under stress. In contrast, Cazzone Giallo seemed to compartmentalize most sodium in the stem. While sodium accumulation in the stems has been shown to protect shoots from sodium toxicity, in pepper and/or in the specific experimental conditions imposed, this strategy was less efficient than chloride exclusion for salt stress tolerance.

## 1. Introduction

Soil and water salinization are increasingly affecting agricultural land [1]. It is estimated that salinization advances at a yearly rate of 10%, with three hectares per minute becoming unsuitable for cultivation [2,3]. A high concentration of salts in the root zone impairs most physiological functions and cellular metabolism, which eventually leads to reduced growth and crop productivity [4]. Therefore, improving plant tolerance to salt stress would be critical to allow crop cultivation in salinized areas [5,6]. Plants may cope, within certain limits, with salt stress by activating a series of physiological responses. These include the control of water and ion homeostasis [7,8], the regulation of sodium and chloride uptake/accumulation via exclusion and compartmentalization mechanisms [6,9], the detoxification of stress-induced reactive oxygen species (ROS) and the hormonal control of multiple stress responses [10,11]. Most research suggests that these mechanisms are less efficient in domesticated plants compared to wild relatives, as well as in commercial cultivars compared to landraces, which were not bred for commercial traits [12,13,14,15]. When compared to a commercial tomato variety, two tomato landraces showed higher salinity tolerance in terms of yield, which was correlated with a higher K^+^/Na^+^ ratio in landraces leaves [16]. The control of ion homeostasis is therefore an important mechanism to overcome salt stress. Similarly, a rice landrace also showed higher salt tolerance when compared to a commercial cultivar [17]. It is generally understood that commercial crops have lost adaptive/resilience traits throughout the breeding process in favor of high-yielding traits [13]. The search for salt-tolerant traits in wild species, and/or minor traditional/landraces has been a quite common approach to rescue such adaptive traits [12]. Sweet pepper (*Capsicum annuum* L.) is one of the most cultivated crops worldwide, and cultivars with different salt tolerances have been reported in previous studies [18,19,20]. However, most reports on sweet pepper describe short-term responses to salinization, a condition that does not reflect the dynamics of seasonal salinization in a field context and may mislead in the identification of useful salt tolerance traits [21]. While short-term exposure to salinity mainly causes an osmotic imbalance, in the long-term, sodium and chloride accumulate in plant tissues, leading to toxicity phenomena [5]. Consequently, plants must activate different tolerance mechanisms for the two phases, whose efficiency may differentiate salt-sensitive vs. salt-tolerant genotypes [22]. Long-term experiments are therefore needed to compare the ability of different species or cultivars/ecotypes to cope with salt stress.

In this work, two sweet pepper genotypes—Quadrato D’Asti (commercial cultivar), the most widespread Italian pepper cultivar, and Cazzone Giallo, a landrace of the Campania Region, Italy—were compared in a long-term experiment to evaluate the physiological basis of their salt stress response. Based on the information collected among local farmers, Cazzone Giallo was considered moderately tolerant to drought. Given that drought and salinity stress share common adaptive traits and that Cazzone Giallo and Quadrato d’Asti had comparable yields, these two varieties were chosen in our analysis. Searching for salt tolerant germplasm is becoming increasingly important since most coastal agriculture of Mediterranean regions, including Southern Italy, are encountering salinization problems [21]. Here, we demonstrated that, compared to the landrace, the commercial cultivar was constitutively more vigorous in terms of leaf area, had a lower yield in the absence of stress, and was more stress tolerant. The high tolerance of the commercial cultivar was associated with a more efficient chloride exclusion mechanism which reduced Cl^−^ accumulation in leaves, thereby allowing the longer functionality of the photosynthetic system.

## 2. Results

### 2.1. Plant Leaf Area, Yield and Chlorophyll Content Index

**Figure 1:**
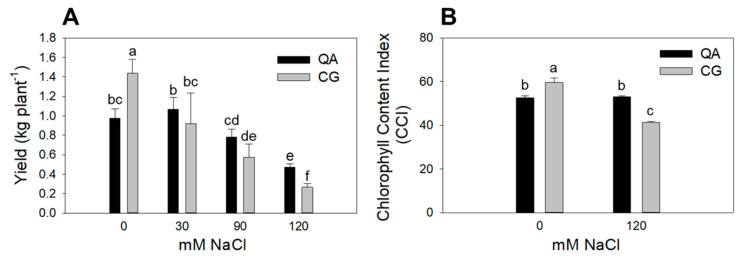
(**A**) Yield (kg plant^−1^, DAT 168) and (**B**) CCI (DAT 65) of the commercial cultivar (QA) and the landrace (CG) grown at control (0 mM), low salinity (30 mM), moderate salinity (90 mM) and high salinity (120 mM) for yield, and at control (0 mM) and high salinity (120 mM) for CCI. Error bars indicate standard error of the means. Means with the same letter are not significantly different according to Duncan’s post hoc test (*p* < 0.05).

Genotype and salinity both affected leaf area (*p* < 0.01 and *p* < 0.001, respectively), whereas there was no interaction between these two factors (Table 1). The landrace had a significantly lower leaf area (−40%) compared to the commercial cultivar. Higher salinity caused a progressive reduction in leaf area at moderate (−73%) and high (−94%) salinity in both genotypes (Table 1). Plant height was similar between the two genotypes, while salinity induced significant a reduction in this parameter, starting at moderate salinity (Table 1). The interaction between genotype and salinity was highly significant (*p* < 0.001) for yield (Table 1). Post hoc analysis showed that the commercial cultivar yield was lower (−32%) than that of the landrace under control conditions. However, upon salinization, the commercial cultivar showed no yield reduction vs. control plants up to the moderate salinity. A significant yield reduction in the commercial cultivar occurred only in the high salinity treatment (−49% vs. control plants). In contrast, salt stress significantly affected the landrace yield at all salinity levels, with −36%, −61% and −82% reductions vs. control plants at low, moderate and high salinity, respectively (Figure 1A, Table 1). The interaction between genotype and salinity also had a significant impact (*p* < 0.001) on the chlorophyll content index (CCI). CCI decreased by 31% in the landrace grown at high salinity, whereas in the commercial cultivar, CCI did not differ from control plants (Figure 1B, Table 1). In addition, the landrace showed a constitutively higher CCI (+12%) compared to the commercial cultivar.

### 2.2. Physiological Parameters

**Figure 2:**
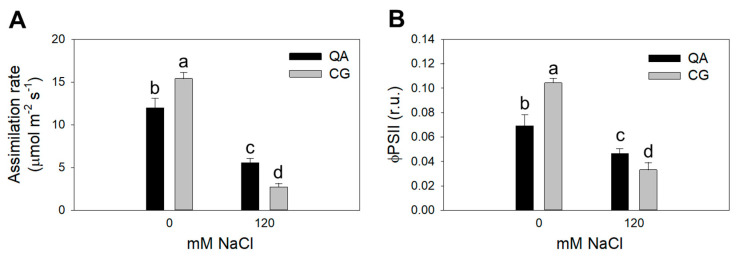
(**A**) Net photosynthetic CO_2_ assimilation (*A*, µmol m^2^ s^−1^), and (**B**) quantum yield of PSII (Φ_PSII_, relative units, r.u.) under 0 mM (control) and 120 mM (high salinity) in the commercial cultivar QA (black bars) and in the landrace CG (gray bars) at DAT 102. Means with the same letter are not significantly different according to Duncan’s test.

Leaf water potential (Ψ_l_) and stomatal conductance (g_s_) were only affected by salinity, while no differences were found with respect to the genotype or the interaction between these two factors (Table 2). In contrast, the interaction between genotype and salinity significantly affected the CO_2_ assimilation rate and quantum yield of photosystem II (Φ_PSII_) (Table 2). The commercial cultivar showed a 22% lower CO_2_ assimilation rate compared to the landrace under control conditions. On the contrary, this response was reverted at the high salinity treatment, with two-fold change higher CO_2_ assimilation rates in the commercial cultivar vs. the landrace (Figure 2A, Table 2). The commercial cultivar maintained a 28% higher Φ_PSII_ than the landrace at high salinity, whereas under control conditions, the commercial cultivar had a 34% lower Φ_PSII_ (Figure 2B, Table 2).

**Figure 3:**
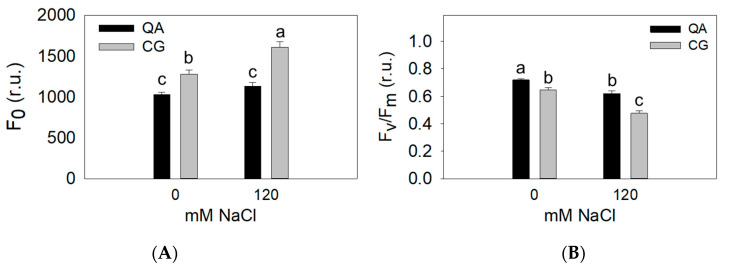
(**A**) Dark-adapted fluorescence emission (F_0_, relative units, r.u.) and (**B**) maximum quantum yield of PSII (F_v_/F_m_, r.u.) of the commercial cultivar (QA) and the landrace (CG) grown under control (0 mM) and high salinity (120 mM) treatments at DAT 133. Means with the same letter are not significantly different according to Duncan’s post hoc test (*p* < 0.05).

The fluorescence emission of dark-adapted leaves was measured in control and high salinity treatments. Under non-salinized control conditions, the landrace showed constitutively higher F_0_ and lower F_v_/F_m_ (Figure 3, Table 3). F_0_ and F_v_/F_m_ were both significantly affected by the interaction between genotype and salinity (Figure 3, Table 3). F_0_ was found significantly higher in the landrace compared with the commercial cultivar at high salinity (+18%) (Figure 3A, Table 3), while F_v_/F_m_ significantly decreased in the landrace compared to the commercial cultivar grown under high salinity (−10%) (Figure 3B, Table 3).

### 2.3. Leaf and Stem Ion Contents

**Figure 4:**
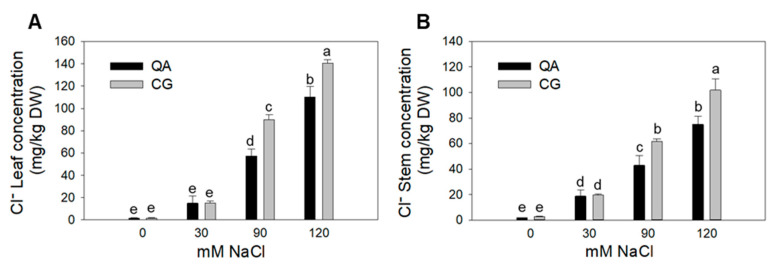
Chloride (mg/kg dry weight, DW) content in leaves (**A**) and stems (**B**) of the commercial cultivar (QA) and the landrace (CG) grown at control (0 mM), low salinity (30 mM), moderate salinity (90 mM) and high salinity (120 mM) at DAT 168. Error bars indicate standard error of the means. Means with the same letter are not significantly different according to Duncan’s post hoc test (*p* < 0.05).

Na^+^ concentration in leaves and stem tissues was not affected by the genotype, yet it significantly increased with salinity (Table 4). In contrast, a significant interaction between genotype and salt treatment was observed in both leaves and stems for Cl^−^ (Figure 4, Table 4).

**Figure 5:**
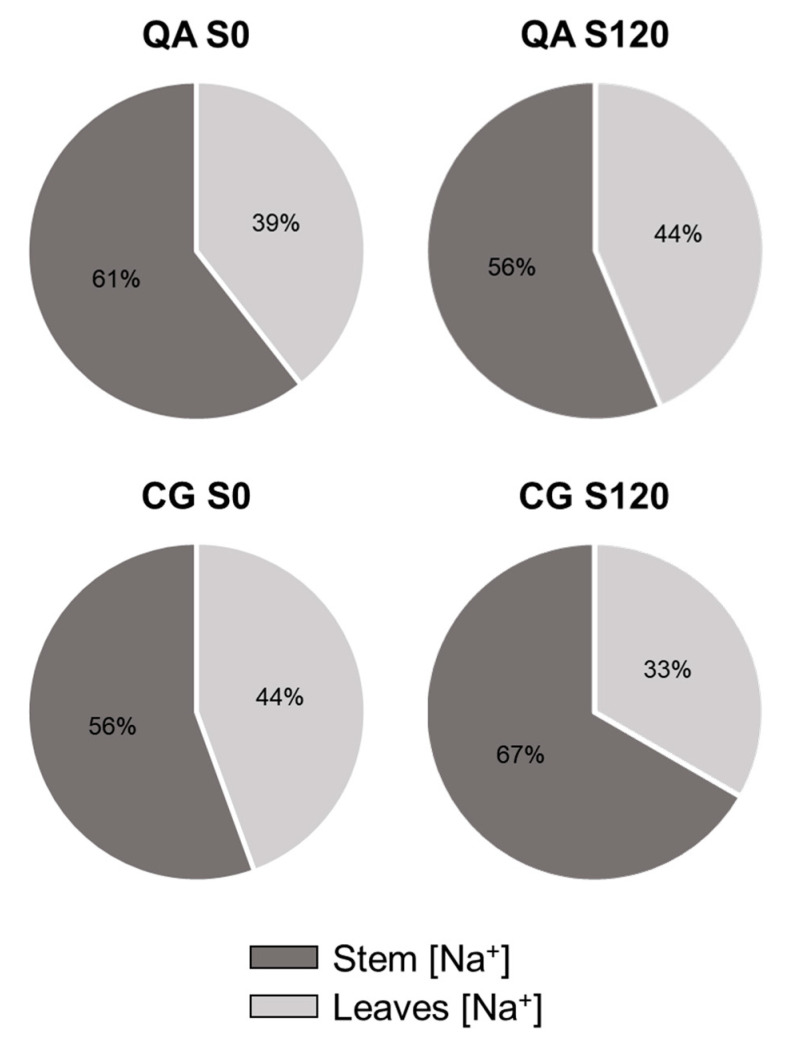
Sodium concentration portioning between leaves and stems of the commercial cultivar (QA) and the landrace (CG) grown at control (0 mM) and high salinity (120 mM) at DAT 168. The number in the pie graph indicates the percentage of sodium found in the different plant portions compared to the total sodium concentration between leaves and stems.

The leaf Cl^−^ concentration in the landrace was 36% and 21% higher than the commercial cultivar at moderate and high salinity, respectively (Figure 4A, Table 4). A similar trend was observed for stems, with 30% and 26% higher concentrations in the landrace than the commercial cultivar at moderate and high salinity, respectively (Figure 4B, Table 4). With respect to Na^+^ and Cl^−^ distribution between stems and leaves, it was found that in the high salinity treatment, stems of the landrace accumulated 50% more Na^+^ than leaves (Figure 5). In contrast, the concentration of Na^+^ in the commercial cultivar grown at high salinity was more balanced between the two organs, with a 21% higher Na^+^ concentration in stems than leaves (Figure 5).

## 3. Discussion

Plants’ ability to overcome salt stress depends on specific adaptation strategies that differentiate tolerant from sensitive species, cultivars or ecotypes. In agricultural contexts, seasonal salt stress develops over time [21], therefore critical adaptation mechanisms aim at (a) reducing water consumption in the first osmotic phase, and (b) improving plant ion homeostasis in the following ionic phase. Plants can best cope with salt stress if they can activate both mechanisms [5]. These plants would be resilient to the harmful effects of salinity in terms of biomass accumulation, potentially resulting in less affected yields. Among different species and cultivars/ecotypes, there is variability in terms of salt stress tolerance [23]. In contrast to high yielding commercial cultivars, traditional ecotypes and/or landraces are used in low input agricultural systems and/or suboptimal environments since they may have conserved stress tolerance traits, which represent an important resource for breeding programs [24,25,26].

Pepper is moderately sensitive to salt stress, showing yield losses when it grows in soils with an electrical conductivity as low as 1.5 dS m^−1^ [27]. Some pepper cultivars have been evaluated for salt stress tolerance [19]. However, their tolerance has been tested only on short-term responses, which do not reflect real agricultural settings [18,19,20], where soil salinization occurs throughout the growing season. Under these conditions, seasonal sodium and chloride accumulation in soil and plants may affect yield and product quality even under moderately saline irrigation [28].

Our results demonstrate that the commercial cultivar (Quadrato D’Asti) maintained a higher yield (Table 1, Figure 1A) and better functionality of the photosystems under salt stress compared to the landrace (Cazzone Giallo) (Table 2, Figure 2). This may have also been the result of a better hydration state of the commercial cultivar, which showed significantly higher leaf water potential values compared to the landrace at early developmental stages (data not shown). This phenomenon has been observed in pepper and is associated with the production of compatible solutes [29]. In this experiment, the different tissue chloride accumulation patterns could also explain the performance of the commercial cultivar and the landrace plants in response to salinity. Chloride concentration was 22–25% lower in both the leaves and stems of the commercial cultivar compared to the landrace when plants were grown under the most severe salt stress (Figure 4). Chloride is needed at micromolar concentrations for plant growth but can be toxic when it accumulates in plant tissues above a certain threshold [9]. In saline soils, plants accumulate high levels of chloride and, in some cases, chloride has been proven to be more dangerous than sodium [30]. Nevertheless, the mechanisms that control chloride accumulation and partitioning in plants have been scarcely addressed [9]. When chloride concentration reaches the toxicity threshold, chloroplast membrane stability and Rubisco efficiency are severely affected [31,32,33]. Consequently, chloride toxicity in plants induces leaf chlorosis and photosynthesis impairment, which in the most sensitive species causes severe growth and yield loss [31,33]. Several studies have identified chloride exclusion as an important adaptive mechanism for plants to cope with salt stress [32,33,34]. Our results show that the commercial cultivar had a higher chlorophyll content (Figure 1B), higher CO_2_ assimilation rate (Figure 2), higher F_v_/F_m_ and lower F_0_ compared to the landrace (Figure 3). The overall higher efficiency of the photosynthetic machinery in the commercial cultivar vs. the landrace was possibly associated with a lower leaf chloride concentration in the former. Considering that Cl^−^ typically follows the transpiration flux [35] and that both cultivars had similar stomatal conductance, the 21% Cl^−^ reduction in the commercial cultivar, on a tissue dry weight basis, indicates that the restriction of Cl^−^ flux to the leaves occurred at the root level [35].

Ion distribution between different organs has also been documented as an effective detoxification strategy under salt stress [36,37]. At the highest salinity, the sodium concentration of the stem in the landrace was twice the leaf sodium concentration, while in the commercial cultivar this ion was almost equally distributed between these organs (Figure 5). Sodium translocation to organs which are less damaged by its build-up, like stems and roots, as well Na^+^ extrusion through salt glands in some halophytes, allows plants to avoid its accumulation in sensitive green tissues [6,38,39]. In leaves, pH alteration and Na^+^/K^+^ competition impairs several metabolic pathways and enzymatic systems related to the photosystem, which will eventually lead to reduced growth rates [4,40]. Although plants’ ability to translocate sodium away from sensitive organs is an important trait associated with salt stress tolerance [39], our results show that this physiological mechanism seems to be more efficient in the less tolerant landrace, whereas it is likely less efficient in the more tolerant commercial cultivar. This suggests that chloride exclusion, rather than sodium partitioning, determined the better performance of the commercial cultivar compared to the landrace when grown under salt stress. In this regard, it is worth noting that plants of the commercial cultivar were much more vigorous in terms of leaf area compared to the landrace (Table 1), yet they were less stressed notwithstanding their greater vigor (Table 1). High vigor, a morphological trait normally associated with high productivity, has generally been gained throughout breeding programs at the expense of stress adaptation [41]. Despite the high vigor of the commercial cultivar, yield stability was also conserved through breeding, since yield was only moderately affected by salt stress. Similar results have been found for strawberry [35], which also had better yield performance in response to salt stress in the cultivar with a larger leaf area. Identifying critical traits that uncouple plant growth and stress responses is therefore pivotal to profile the genetics of stress adaptation in different crops/varieties and overall to improve crop stress resilience [42].

Overall, our results indicate that larger leaf area and the control of chloride partitioning among tissues/organs are important traits for salt stress tolerance in sweet pepper. Although we cannot establish a clear functional link between leaf area expansion and ion detoxification mechanisms, an efficient chloride exclusion system could be a salt stress tolerance determinant that may preserve the photosynthetic machinery and allow biomass accumulation (larger leaf area), which in turn would sustain yield in saline environments. Chloride detoxification may be more important than sodium detoxification in sweet pepper, as previously reported for other species [33,37]. However, the landrace ability to translocate sodium to the stem could also be an effective strategy in environments in which sodium is the main environmental stressor, such as saline–sodic soils, as also reported [41]. The effectiveness of these responses could have also been affected by the constitutive partitioning of photosynthates in the absence of stress, which was in favor of the vegetative organs in the commercial cultivar vs. reproductive organs in the landrace.

## 4. Conclusions

We report here that the commercial cultivar Quadrato D’Asti performed better than the landrace Cazzone Giallo when subjected to long-term salt stress. Chloride control in leaves seemed to be the main mechanism for the higher tolerance of the commercial cultivar compared to the landrace, which provides interesting insights into the mechanisms that sweet pepper plants use to cope with salt stress. A more efficient chloride control system proved to be more effective for sweet pepper tolerance to salinity than sodium portioning in the specific experimental conditions imposed. Indeed, the commercial cultivar maintained higher growth and productivity than the landrace when subjected to salt stress thanks to the longer functionality of the photosystem with increasing salinity. Comparative analysis of salt stress response and tolerance in several pepper cultivars/ecotypes may help in elucidating the role of Na^+^ and Cl^−^ control in salt-sensitive vs. salt-tolerant crops. Furthermore, unraveling the relationship between plant vigor and yield between the two genotypes would also be critical to fully understand the trade-off between growth and stress adaptation traits in agricultural crops.

## 5. Materials and Methods

### 5.1. Plant Material, Growth Conditions and Salt Treatments

Two sweet pepper genotypes, Quadrato D’Asti (QA, commercial cultivar) and Cazzone Giallo (CG, landrace) were tested for their ability to tolerate salt stress. In the Campania region (Southern Italy), Cazzone Giallo was one of the most common landraces in the past, but it has been replaced by the Quadrato D’Asti, a commercial cultivar now grown all over Italy. The experiment was carried out in a greenhouse of CREA-OF (Pontecagnano, SA, Italy) with an automatic, computer-controlled soilless system equipped with a drip fertigation device. A basic nutrient solution with minor modifications [43] was used as control treatment (0 mM). The nutrient solution was supplemented with NaCl to obtain low (30 mM), moderate (90 mM) and high (120 mM) salinity. The electrical conductivity (EC_w_) of the four solutions was 2.6, 5.8, 12.4 and 15.6 dS m^−1^, respectively. Both the pH and EC_w_ of each solution were controlled daily and kept constant through the whole growing crop cycle. The pH was kept within the 5.5–6.0 range by adding HNO_3_, whereas EC_w_ was stabilized by adding fresh water to the nutrient solution.

Seeds of the commercial cultivar Quadrato D’Asti and the landrace Cazzone Giallo were provided by SAIS S.p.A (Cesena, Italy) and Arca 2010 (Acerra, NA, Italy), respectively. Seeds of both genotypes were germinated in a greenhouse at Arca 2010 in 220-hole alveolar trays containing peat. The transplant was performed 37 days after seeding when the plantlets reached approximately 15 cm high in a 27 cm diameter pot with one seedling per pot. One hundred and twenty plants, 60 plants per genotype, were distributed in the four saline treatments (15 plants per treatment). The salt treatment started 13 days after transplanting (DAT 13) and continued until the “yellow fruit” stage (DAT 168). Each of the four solutions was pumped from an independent tank to the corresponding channel where each of the 30 pots (15 pots per genotype) was provided with two emitters (emitter flow rate: 2 L h^−1^). The amount of the supplied nutrient solution was on average 2.25 L plant^−1^ day^−1^ with a daily consumption per plant ranging from 0.038 to 1.0 L for the control, 0.031 to 0.842 L for low salinity, 0.038 to 0.45 L for moderate salinity and 0.038 to 0.385 L for high salinity. The solution drained from the pot was returned to its tank for later recirculation (closed system). The high ratio between the supplied and consumed nutrient solution made it possible to have the solution sampled in the root zone with the same composition as the recirculated solution in the tank (data not shown). During the experiment, the greenhouse temperature ranged from 18 to 30 °C and relative humidity from 60 to 85%.

### 5.2. Growth Measurements and Yield

The leaf area (LA, cm^2^) was measured in three collected replicate plants per treatment by means of an electronic area meter (Li-3100, LICOR Lincoln, NE, USA). Fruit yield (kg plant^−1^) was measured in six replicate plants per treatment. LA and yield were assessed at harvest (DAT 168).

### 5.3. Plant Water Status

Total leaf water potential (Ψ_l_, MPa) was assessed at 102 DAT between 10:00 to 12:00 on a top fully expanded and light-exposed leaf of three replicates per genotype in plants grown at control (0 mM) and high salinity (120 mM) by means of a Scholander-type pressure chamber (SAPS II, 3115, Soil moisture Equipment Corp., Santa Barbara CA, U.S.A). After cutting, the leaf was inserted into the pressure chamber within not more than 30 s, and the pressure was increased at a rate of 0.2 MPa min^−1^.

### 5.4. Leaf Gas Exchanges and Modulated Chl a Fluorescence

Net photosynthetic CO_2_ assimilation rate (A, µmol m^−2^ s^−1^) at saturating light, stomatal conductance to water vapor (g_s_, mol m^−2^ s^−1^) and leaf temperature were determined using a portable open-system gas-exchange analyzer Li-6400XT (Li-Cor Biosciences, Lincoln, NE, USA), with CO_2_ inside the leaf chamber set to 400 µmol mol^−1^ air by means of an external bottled CO_2_ source. An LED light source with emission peaks centered at 630 nm in the red light and at 460 nm in the blue light provided a photosynthetic photon flux density (PPFD) equal to 2000 µmol (photons) m^−2^ s^−1^ (90% red, 10% blue). The same instrument provided by built-in fluorometer to measure the modulated fluorescence parameters. The measuring beam was set at intensity five (according to the instrument manual) with a modulation of 20 kHz. After the measurement of Chl *a* fluorescence emission at steady-state under light conditions (F’), the maximum fluorescence emission (F_m_’) was assessed upon induction by a 0.8 s super-saturating light pulse at 6000 µmol (photons) m^−2^ s^−1^, with a modulation of 20 kHz. The software of the instrument calculated the gas-exchange parameters based on the von Caemmerer and Farquhar model [44], and the actual quantum yield (ϕ_PSII_) or ΔF/F_m_’ = (F_m_’ − F’)/F_m_’ [45]. Measurements were at DAT 102 between 10:00 to 12:00 on a top fully expanded and light-exposed leaf in eight replicates per genotype in plants grown at control (0 mM) and high salinity (120 mM).

### 5.5. Transient Chl a Fluorescence Emission

The dark-adapted (basal) fluorescence emission (F_0_) and the maximum quantum yield of PSII photochemistry (F_v_/F_m_) were measured by a continuous excitation Handy PEA fluorometer (Hansatech; Instruments King’s Lynn, UK). For fluorescence induction, the instrument adopts an excitation light pulse emitted by a (red) 650 nm light diode source, applied for 1 s at the maximum available PPFD of 3500 µmol (photons) m^2^ s^−1^. Leaves were dark-adapted for 30 min using the equipped white leaf clips, prior to the assessment of F_0_ and maximum Chl *a* fluorescence emission, F_m_, from which the dark-adapted maximum quantum yield of PSII photochemistry was calculated as F_v_/F_m_ = (F_m_ − F_0_)/F_m_ [46]. Measurements were taken at DAT 133 in the morning (10:00–11:00) on two leaves per plant in 12 replicates per genotype on plants grown at control (0 mM) and high salinity (120 mM).

### 5.6. Chlorophyll Content Index (CCI)

At DAT 65, an optical meter (SPAD-502, Minolta, Japan) was used to measure the leaf chlorophyll content index (CCI, r.u.) [47] on six replicates per genotype on plants grown at control (0 mM) and high salinity (120 mM).

### 5.7. Leaf and Stem Ion Content

At the end of the experiment (DAT 168), stems and leaves of the three plants per treatment were separately sampled for the quantification of Na^+^ and Cl^−^ content by means of ion chromatography using a Dionex ICS-3000 (Sunnyvale, CA, USA) [48] on three replicate plants.

### 5.8. Statistical Analyses

We used two-way ANOVA with genotype, salinity and their interaction as the independent factors with the null hypothesis rejected at the *p* < 0.05 significance level. Mean separations were performed by Duncan’s post hoc test using IBM SPSS Statistics 21 (Armonk, NY, USA).

## Figures and Tables

**Table 1:** Effect of genotype, salinity and their interaction on leaf area, height and yield at 168 days after transplant (DAT) and chlorophyll content index (CCI, DAT 65) of the commercial cultivar (QA) and the landrace (CG) grown at control (0 mM), low salinity (30 mM), moderate salinity (90 mM) and high salinity (120 mM). ns, **, *** indicate not significant or significant differences at *p* < 0.01 and *p* < 0.001, respectively, according to ANOVA. In each column, for each factor, means with the same letter are not significantly different according to Duncan’s post hoc analysis (*p* < 0.05).

Factors	LA	Height	Yield	CCI
	(cm^2^)	(m)	(kg Plant^−1^)	(r.u.)
**Genotype (G)**	**	ns	ns	ns
**QA**	4067 a	0.76	0.82	52.9
**CG**	2462 b	0.79	0.80	50.5
**Salinity (S)**	***	***	***	***
**S_0_**	5648 a	0.92 a	1.20 a	56.1 a
**S_30_**	4949 a	1.00 a	0.99 b	-
**S_90_**	1565 b	0.64 b	0.68 c	-
**S_120_**	896 b	0.53 c	0.37 c	47.2 b
**G × S**	ns	ns	***	***
**QA S_0_**	6889	0.85	0.97 bc	52.6 b
**CG S_0_**	4408	1.00	1.44 a	59.6 a
**QA S_30_**	6163	1.00	1.06 b	-
**CG S_30_**	3734	1.01	0.92 bc	-
**QA S_90_**	1867	0.64	0.782 cd	-
**CG S_90_**	1263	0.64	0.575 de	-
**QA S_120_**	1347	0.56	0.473 ef	53.1 b
CG S_120_	444	0.51	0.266 f	41.3 c

**Table 2:** Effect of genotype, salinity and interaction on leaf water potential (Ψ_l_), stomatal conductance (gs), net photosynthetic CO_2_ assimilation (A, µmol m^2^ s^−1^) and Φ_PSII_ at DAT 102 of the commercial cultivar (QA) and the landrace (CG) grown at control (0 mM) and high salinity (120 mM). ns, **, *** indicate not significant or significant differences at *p* < 0.05, *p* < 0.01 and *p* < 0.001, respectively. In each column, for each factor, means with the same letter are not significantly different according to Duncan’s post hoc test (*p* < 0.05).

Factors	Ψ_l_	g_s_	A	Φ_PSII_
	(MPa)	(mol m^−2^ s^−1^)	(µmol m^−2^ s^−1^)	(r.u.)
**Genotype (G)**	ns	ns	ns	ns
**QA**	−0.91	0.19	8.78	0.060
**CG**	−0.92	0.16	8.38	0.063
**Salinity (S)**	***	***	***	***
**0 mM**	−0.62 a	0.37 a	13.42 a	0.084 a
**120 mM**	−1.25 b	0.05 b	4.15 b	0.040 b
**G × S**	ns	ns	***	**
**QA S_0_**	−0.58	0.36	12.00 b	0.069 b
**CG S_0_**	−0.65	0.38	15.41 a	0.104 a
**QA S_120_**	−1.27	0.06	5.57 c	0.046 c
**CG S_120_**	−1.23	0.04	2.72 d	0.033 d

**Table 3:** Effect of genotype, salinity and interaction on basal emission (F_o_) and on maximal quantum yield of PSII (F_v_/F_m_) at DAT 133 of the commercial cultivar (QA) and the landrace (CG) grown at control (0 mM) and high salinity (120 mM). ns, *, *** indicate not significant or significant differences at *p* < 0.05, *p* < 0.01 and *p* < 0.001, respectively. In each column, for each factor, means with the same letter are not significantly different according to Duncan’s post hoc test (*p* < 0.05).

Factors	F_0_	F_v_/F_m_
	(r.u.)	(r.u.)
**Genotype (G)**	***	***
**QA**	1083 b	0.67 a
**CG**	1445 a	0.56 b
**Salinity (S)**	***	***
**0 mM**	1156 b	0.68 a
**120 mM**	1371 a	0.55 b
**G × S**	*	*
**QA S_0_**	1031 c	0.72 a
**CG S_0_**	1281 b	0.65 b
**QA S_120_**	1134 c	0.62 b
**CG S_120_**	1269 a	0.48 c

**Table 4:** Effect of genotype, salinity and their interaction on Na^+^ and Cl^−^ content in leaves and stems of the commercial cultivar (QA) and the landrace (CG) grown at control (0 mM), low salinity (30 mM), moderate salinity (90 mM) and high salinity (120 mM) at DAT 168. ns, *, **, *** indicate not significant or significant differences at *p* < 0.05, *p* < 0.01 and *p* < 0.001 according to ANOVA, respectively. In each column, for each factor, means with the same letter are not significantly different according to Duncan’s post hoc analysis.

Factors	Leaves		Stem	
	Na^+^	Cl^−^	Na^+^	Cl^−^
	(mg kg^−1^ DW)			
**Genotype (G)**	ns	***	ns	**
**QA**	6.1	46.0 b	9.1	34.6 b
**CG**	4.9	61.8 a	11.9	46.5 a
**Salinity (S)**	***	***	***	***
**0 mM**	0.4 b	1.7 d	0.6 c	2.3 d
**30 mM**	3.7 b	15.0 c	9.0 b	19.2 c
**90 mM**	6.1 b	73.5 b	13.6 ab	52.3 b
**120 mM**	11.7 a	125.4 a	19.0 a	88.4 a
**G × S**	ns	*	ns	*
**QA S_0_**	0.39	1.6 e	0.6	1.9 e
**CG S_0_**	0.48	1.7 e	0.6	2.7 e
**QA S_30_**	3.6	15.0 e	10.4	18.7 d
**CG S_30_**	3.8	15.0 e	7.5	19.7 d
**QA S_90_**	8.0	57.3 d	9.6	42.9 c
**CG S_90_**	4.2	89.7 c	17.7	61.7 b
**QA S_120_**	12.4	110.1 b	16.0	75.0 b
**CG S_120_**	11.0	140.6 a	22.0	101.9 a
